# Alexithymia modulates the attitudes towards odors but not the olfactory abilities or the affective reactions to odors

**DOI:** 10.1371/journal.pone.0278496

**Published:** 2023-06-06

**Authors:** Cinzia Cecchetto, Elisa Dal Bò, Marilena Aiello, Florian Ph. S. Fischmeister, Claudio Gentili, Sofia Adelaide Osimo

**Affiliations:** 1 Department of General Psychology, University of Padua, Padua, Italy; 2 Padova Neuroscience Center (PNC), University of Padua, Padua, Italy; 3 Department of Psychology “Renzo Canestrari”, University of Bologna, Bologna, Italy; 4 Department of Biomedical Imaging and Image-Guided Therapy, Medical University of Vienna, Vienna, Austria; 5 Institute of Psychology, University of Graz, Graz, Austria; 6 BioTechMed-Graz, Graz, Austria; 7 Department of Psychology, MibTec, Università degli Studi di Milano-Bicocca, Milan, Italy; Federal University of Paraiba, BRAZIL

## Abstract

Although emotion and olfaction are closely linked, only a few studies have investigated olfactory processing in alexithymia, a condition characterized by altered emotional processing. These results do not allow comprehensive conclusions on whether individuals with alexithymia present lower olfactory abilities or only altered affective reactions and awareness of odors. Three pre-registered experiments were conducted to clarify this relation. We assessed olfactory functions, the affective qualities of odors, the awareness of odors, the attitudes towards them, and the ability to form olfactory images in the mind. Bayesian statistics were used to assess differences between low, medium and high alexithymia groups, and Linear Mixed Models (LMMs) were applied to investigate the modulation of the affective and cognitive components of alexithymia. We observed that individuals with a high level of alexithymia presented the same olfactory abilities, and did not show differences in their rating of odors compared to individuals with low alexithymia levels, while they reported lower levels of social and common odor awareness and a more indifferent attitude towards odors. Olfactory imagery was not affected by alexithymia level, and the affective and cognitive components of alexithymia, when considered separately, modulated olfactory perception differently. Learning more about olfactory perception in individuals with alexithymia leads to a better understanding of how alexithymia impacts the perception of hedonic stimuli coming from different sensory modalities. Our results imply that treatment goals for alexithymia should be the enhancement of the conscious perception of odors, supporting the use of mindfulness-based protocols in the alexithymia treatment.

## Introduction

Alexithymia is a psychological construct characterized by difficulties in identifying, analyzing, and verbalizing feelings, restricted imaginal capacities, and limited emotional experience [1,2]. Although alexithymia co-occurs with a broad range of psychiatric and neurological disorders [3–7] it also has a prevalence rate of 10% in the general healthy population [8]. Indeed, several studies have revealed that even in healthy individuals alexithymia caused alterations in the processing of emotional stimuli [9], such as reduced affective priming [10], reduced emotional facial expressions’ recognition [11–14], and altered physiological arousal to emotional stimuli [15–17]. Alexithymia has been associated with structural brain changes, such as diminished volume of the left insula, left amygdala, orbital frontal cortex and striatum [see 18 for a meta-analyses]. Various studies have also shown that alexithymic individuals, compared to non-alexithymic individuals, present diminished brain activation during emotional processing, such as in the amygdala, which suggests reduced emotional attention [19,20], as well as decreased activation in the right insula and precuneus suggesting reduced emotional awareness [19,21].

Among sensory stimuli, odors are the ones with the strongest link to emotions [22,23]. Olfaction is intimately connected to the limbic system, which is devoted to human emotional processing [24–28]. In addition, hedonic valence is the primary perceptual feature of an odor and the first aspect used to discriminate and describe it [29]. Due to this intimate connection between the olfactory and limbic systems, the sense of smell highly influences everyday life [30,31]; odors affect food selection [32], mood [22,33,34], behavior [35,36], memory [37], and social interactions [38–40]. Despite the preferential link between odors and emotions, only a few studies investigated olfactory perception in individuals with alexithymia (for a review see [41]). These studies, which have often involved individuals with other neurological or psychiatric conditions, present mixed results: one study showed reduced olfactory abilities in individuals with alexithymia [42], while another found no differences or even higher perceived affective qualities of odors in alexithymia [43]. Only one study so far has evaluated the effect of alexithymia on olfactory abilities in healthy individuals. This study showed that while no differences emerged in basic olfactory abilities, alexithymic individuals exhibit altered physiological responses to odors [44]. Moreover, in this study, the affective dimension of alexithymia, which refers to the level at which an individual subjectively experiences emotions [45], was specifically associated with reduced olfactory imagery, while the cognitive dimension, which refers to the level at which an individual is able to verbalize, identify, and analyze emotions, was associated with lower awareness of odors in the environment. The inconsistency of the available results may be the consequence of several factors, such as the heterogeneity of the experimental groups, designs, and olfactory measures (e.g., basic olfactory abilities, affective reactions to odors, and odor modulation on cognitive processes).

Given the relevant differences among the different olfactory measures and the wide differences of domains that olfaction can modulate, the aim of the present study was to clarify whether olfaction is altered in alexithymic individuals through a comprehensive evaluation of odor perception. Specifically, we present here three pre-registered experiments, each of which focused on one specific aspect of olfactory perception. In Experiment 1, we assessed whether individuals with alexithymia presented *lower olfactory functions*, meaning lower absolute sensitivity to the presence of an odor (threshold), and lower ability in recognizing (identification) and discriminating between odors (discrimination). In Experiment 2, the perceived *affective qualities of odors*, meaning the perceived intensity, pleasantness, and familiarity of odors, were compared between alexithymic and non-alexithymic healthy individuals. Finally, in Experiment 3, the *olfactory meta-cognitive abilities* were investigated, meaning how alexithymic and non-alexithymic individuals differed in their *awareness of odors* in the environment [46,47], in their *attitudes towards odors* (i.e., how much they let good or bad smells impact their liking of new foods, new places, new cosmetic/health products and new persons; [48]), and in the *ability to form olfactory images* in their mind [49,50].

Based on previous results, we predicted that individuals with alexithymia would have the same level of olfactory abilities, and rate odors as pleasant, intense, and familiar as non-alexithymic individuals, but that they would report a lower level of olfactory meta-cognitive abilities compared to non-alexithymic individuals.

## Materials and methods

### Participants

Data were collected during larger protocols held at the University of Graz (Austria), the University of Padova (Italy), and SISSA (Trieste, Italy) between October 2017 and November 2020. The protocols were approved by the local ethics committee (SISSA: Protocol n° 3206-III/13; University of Padova: Protocol n° 3667; University of Graz: Protocol n° 39/56/63 ex 2016/17) in accordance with the 1975 Declaration of Helsinki and its later amendments. Data from unrelated projects were aggregated, and then predictions, exclusion criteria, experimental designs and analyses were preregistered at the Open Science Framework (OSF, https://osf.io/vkgcb) before the conduction of the analyses related to the research plan. As stated in the preregistration, since the data had already been collected from different research centers, we could not choose our sample size. Each experiment included a different sample of participants. In Exp 1 and 2, all participants provided written informed consent before participating in the study. In Exp 3, participants accepted informed consent before starting an online survey. In Exp 1 and 2, exclusion criteria were being pregnant, having cardiovascular, neurological, or psychiatric diseases, drug and nicotine consumption, olfactory dysfunction, previous head trauma leading to unconsciousness, chronic rhinosinusitis, and being younger than 18 years and older than 45 years. In Exp 2, only women were included. In Exp 1 and 2, participants with TDI below 16.5 were excluded from the analyses. In addition, it was decided that for Exp 2 participants with hyposmia (TDI score below 30.5) would be excluded from the sample if results from Exp 1 indicated that olfactory abilities do not depend on alexithymia level, to exclude effects of olfactory dysfunctions. In Exp 2 and 3, participants having a BDI-II score above or equal to 20 were excluded, being a possible indication of moderate depression [51]. Moreover, because of the strong association between symptoms of depression and abnormal odor perception [52,53], to further control for this modulation, the BDI-II score was also used as a covariate in Exp 2 and 3.

For Exp 3, the survey was shared via social media and direct emails to targeted Italian or German native speakers between 18 and 45 years old, to minimize variability due to olfactory decline [54].

### Procedure

All participants completed the Bermond–Vorst Alexithymia Questionnaire, form B (BVAQ-B; [55]). The BVAQ-B consists of 20 items rated on a 5-point scale with a total score ranging from 20 to 100, measuring the cognitive (COG; identifying, analyzing, and verbalizing feelings), and the affective dimension (AFF; fantasizing and emotionalizing) [45,56] of alexithymia; participants with a score above 53 are considered alexithymic [57]. Internal reliability (Cronbach’s alpha) for the affective and cognitive subscale ranged from 0.80 to 0.90 [58]. Only for the Bayesian analyses, participants were divided in each experiment into three groups based on the BVAQ proposed cut-off scores [57,59]: Low Alexithymia (LA, BVAQ scores < 43); Medium Alexithymia (MA, 43 ≤ BVAQ ≤ 52) and High Alexithymia (HA, BVAQ > 52). None of the recruited participants was aware of their alexithymia level, nor the purpose of the study.

In Exp 1 and 2, participants were tested for olfactory function using the computer-testing version of the standardized “Sniffin’ Sticks” test (Burghart Instruments, Wedel, Germany; [60]), which assessed odor identification, odor detection threshold, and odor discrimination. Individuals with a total TDI (Threshold Discrimination Identification) score below 16.5 were considered anosmic [61]. See the Supplementary material for details regarding the test and differences among experiments. In Exp 2, during the identification test, participants were asked to rate each odor for intensity, pleasantness, and familiarity after having answered the identification question. Ratings were collected on a 10-point computerized Visual Analogue Scale (VAS), ranging from “not at all” to “very much”. Participants also rated how hungry they were on a 10-point computerized VAS ranging from “not at all” to “very much” to explore whether the hunger state affects the hedonic perception of odors and completed the Beck Depression Inventory-II (BDI-II; [51]; Italian version by [62], German version by [63]).

In Exp 3, the survey began with questions regarding demographic information (age, gender). Since this study did not explore real olfactory abilities but olfactory meta-cognitive abilities, smokers were not excluded, but participants were asked to state if they were smokers and how many cigarettes they smoked per day. Participants then completed questionnaires regarding olfactory meta-cognitive abilities: the Olfactory Awareness Scale (OAS; [46,64]); the Social Odor Scale (SOS; German and Italian version by [47]); the Affective Importance of Odor scale (AIO; [48]); and the Vividness of Olfactory Imagery Questionnaire (VOIQ; [65]). OAS, AIO VOIQ were first translated from the English version in German and Italian by bilingual speakers and, second, revised and approved by two experts for a previous study [47]. Finally, they completed the BDI-II [51] (Italian version by [62]), and the BVAQ-B [55].

### Questionnaires

OAS and SOS are two questionnaires measuring odor awareness. The OAS [46] is a 34-item questionnaire that evaluates a person’s tendency to pay attention to odorants in the environment. Its total score is calculated by the addition of the items (ranging from 32 to 158), with higher scores indicating higher odor awareness. Cronbach’s alpha for the OAS scale ranged between 0.77 and 0.80 [46]. The SOS [47] measures the awareness of social odors (i.e. body odors) in everyday life. It is composed of 12 items rated on a 5-point scale, with a total score ranging from 0 to 48, with higher scores indicating higher attention toward social odors. The internal consistency (McDonald’s ω) of the SOS ranged between 0.70 and 0.80 [47]. The AIO [48] is an 8-item questionnaire measuring the type of attitudes individuals show towards odors, i.e. how much they let good or bad smells impact their liking for new foods, new places, new cosmetic/ health products and new persons. Cronbach’s alpha for the AIO scale ranged between 0.73 and 0.75 [48]. The VOIQ [65] measures olfactory imagery, participants are asked to mentally evoke 16 odors and activities associated with odors (e.g. preparing barbeque) and to estimate the vividness of each of the evoked odors using a 5-point scale (“1—No odor at all, you only know that you are thinking of the odor” to “5—perfectly realistic and as vivid as the actual odor”).

### Statistical analyses

Predictions and statistical analyses were pre-registered at the Open Science Framework (OSF, https://osf.io/vkgcb). We first analyzed these data using a Bayesian approach to enable the assessment of the null hypothesis; then, we also applied an exploratory approach consisting of a series of Linear Mixed Models (LMMs) to determine the modulation of the two components of alexithymia on the different aspects of olfactory perception. Data were cleaned and analyzed using the software R. Descriptive analyses of the experimental measures were run using *t-tests* (*stats* package; R Core [66]). All continuous variables were centered and scaled so that their mean equals zero and their SD equals one. Bayesian statistics were implemented using JASP software [67,68] which calculates Bayes factors (BF). The subscript in the Bayes factor notation indicates which hypothesis is supported by the data: BF_10_ indicates the Bayes factor in favor of H1 (presence of differences between groups based on BVAQ) over H0 (no differences between groups), whereas BF_01_ indicates the Bayes factor in favor of H0 over H1. Indeed, BF_10_ = 1/BF_01_, where larger values of BF_10_ indicate more support for H1 [68]. In particular, BF values were interpreted following standard recommendations [69,70]: BF between 1 and 3 implies indecisive to anecdotal evidence, 3–10 substantial, and 10–30 strong evidence. Exploratory analyses consisted of a series of Linear Mixed Models (LMMs) implemented using the software R and computed for each dependent variable using the *lmer* function (lme4 package, [71]) and explored using the *anova* type 3 function of the car package [72]. To ensure that each predictor improved the models’ fit, models were simplified using the *step* function (lmerTest package, [73]), which relies on the AIC criterion [74]. Factors that did not significantly improve the models’ fit were removed. AIC values of the initial and final models were calculated using the *anova* function (stats package, [66]). When the random factors did not improve the model’s fit, they were removed and the model was rerun using the function *lm* (stats package, [66]). To explore post-hoc interactions between continuous factors we used the *sim_slopes* function of the interactions package [75]. Collinearity between predictors was measured by calculating the Variance Inflation Factors (VIF) with the *vif* function of the car package [72]. All factors showed low collinearity, with values below 2. See Supplementary material for information on the final LMMs predictors and Tables reporting zero-order correlations between all factors (S1, S2 and S3 Tables in S1 File).

Normality of the residuals of both the Bayesian analyses and the LMMs was checked through visual inspection of the q-q plots (quintile-quintile). Significant deviations from linearity of the observations or non-symmetric scales indicated a deviation from normality of the residuals. In case of significant deviation, values with more distant than plus or minus 3 Median Absolute Deviation (MAD) from the median were removed.

In Exp 1, four Bayesian Analyses of Covariance (ANCOVA) were performed separately for TDI scores and subtest scores (Threshold, Discrimination, and Identification), with groups based on BVAQ scores as fixed factors and language and gender as random factors. Default priors (r scale fixed effects = 0.5; r scale random effects = 1; r scale covariates = 0.35) were applied [76]. LMMs were run for TDI and subtest scores (Threshold, Discrimination, and Identification). The predictors consisted of the main effects and the interactions of the affective and the cognitive components of the BVAQ scale, plus the main effects of age and gender, which were used as covariates. Initial models also included language as a random factor (Italian/German):

∼AFF*COG+Age+Gender+(1|Language)

In Exp 2, Bayesian analyses were performed on Sniffin’ Sticks test scores, first to evaluate whether the results of Exp 1 were confirmed and then on the perceptual qualities of odors (intensity, pleasantness, and familiarity). Averages of intensity and familiarity ratings were calculated across all odorants. Averages of pleasantness ratings were calculated separately for unpleasant (turpentine, garlic, and fish), neutral (shoe leather, liquorice, coffee, and clove), and pleasant odorants (orange, cinnamon, peppermint, banana, lemon, apple, pineapple, rose, and anise). These classifications were based on the results of the database Hedos-F [77–79]. Test modality (classical procedure/single-use paper strips; see Supplementary material) and BDI-II were added as covariates.

LMMs were computed for Intensity, Pleasantness, and Familiarity ratings. The predictors consisted of the main and interaction effects of the affective and cognitive components of the BVAQ scale and of the BDI scores; in addition, as most of the odors presented in Exp 2 were food odors, we explored the interaction between each of these three predictors with the individual hunger level and with a dichotomic variable indicating whether the odor rated was a food or a non-food odor. Age was used as a covariate and the test modality and participant ID were included as random factors. Again, pleasantness ratings were analysed separately for unpleasant, neutral, and pleasant odorants:

∼AFF*COG*BDI+AFF*food/non‐food*Hunger+COG*food/non‐food*Hunger+BDI*food/non‐food*Hunger+Age+(1|Modality)+(1|ID)

In Exp 3, Bayesian analyses were performed on OAS, SOS, AIO, and VOIQ. BDI-II was added as a covariate. LMMs were computed for each dependent variable (OAS, SOS, AIO, and VOIQ) with the main and interaction effects of the affective and the cognitive components of the BVAQ scale and the BDI-II scores as predictors; in addition, age, gender, and smoking habits were used as covariates and the language of the participants was included as a random factor:

∼COG*AFF*BDI+Age+Gender+Smoking+(1|Language)

Datasets and R code are available here https://osf.io/q5rmt/.

## Results

### Samples characteristics

Exp 1 included an initial sample of 198 participants. Eleven participants were removed because of anosmia (TDI < 16.5). The final sample included 187 participants. Of these, 81 were native German speakers, and 106 were native Italian speakers.

Exp 2 included an initial sample of 172 women. Bayesian analyses on the Sniffin’ Sticks test (TDI score and sub-scores) showed strong evidence for the null hypothesis that olfactory abilities do not depend on alexithymia level, confirming the results of Exp 1. According to the exclusion criteria, 25 participants were removed because of BDI-II scores equal to or higher than 20, and, as the results of Exp 1 showed strong evidence that olfactory abilities do not depend on alexithymia level, participants with a TDI score below 30.5 (N = 23), indicating hyposmia, were excluded from the sample. The final sample comprised 124 participants [Italian women between 18 and 45 years old (mean = 22.92, SD = 2.46)], 29 of whom performed the “Sniffin’ Sticks” test with the classical procedure, and 95 that performed it with single-use paper strips. No significant differences were found between these two testing modalities for the TDI score [t(122) = 1.48, *p* = 0.14], identification score [t(122) = 0.62, *p* = 0.53], detection score [t(122) = 0.33, *p* = 0.74], or threshold score [t(122) = 1.26, *p* = 0.21].

In Exp 3, from the starting sample size (649 individuals), 110 participants were removed because they did not answer correctly to the 4 control items included to control the tendency of some participants to answer items without reading the content [80] and to control for respondents’ inattention which may have been particularly high due to the pandemic situation [81]. 220 participants were removed due to one of the following exclusion criteria: pregnancy or breastfeeding, presence of somatic, neurological, or psychiatric conditions, diagnosis of COVID-19 (Parma et al., 2020), and failure to report gender. Moreover, we removed 46 participants whose BDI-II scores were above or equal to 20 (moderate depression) for a final sample size of 383 participants. The final sample size included 292 women and 91 men between 18 and 45 years old (mean = 24.03, SD = 5.06). Of these, 128 were native German speakers and 255 were native Italian speakers. Eighty-four participants reported to be smokers but they were equally distributed across the three alexithymic groups (HA = 20, LA = 29, MA = 35; χ^2^(2) = 3.12, p = 0.21).

Please refer to Table 1 for details on the groups’ characteristics.

**Table 1:** Summary table of group characteristics.

	HA Mean (SD)	MA Mean (SD)	LA Mean (SD)	Range
**Exp 1**	58	75	54	-
F:M	45:13	64:11	43:11	-
Age	24.69 (4.83)	23.73 (4.28)	26.12 (5.65)	18–44
BVAQ	57.87 (4.33)	47.69 (2.65)	37.66 (4.33)	26–69
AFF	23.26 (3.87)	18.83 (4.41)	16.01 (3.89)	9–34
COG	34.62 (5.18)	28.87 (4.67)	21.65 (4.38)	13–46
TDI	34.03 (5.09)	33.93 (4.57)	34.22 (4.30)	17–46
Threshold	7.36 (3.19)	7.73 (3.23)	7.41 (3.06)	1–16
Discrimination	12.93 (2.02)	12.97 (1.83)	13.03 (2.26)	5–16
Identification	13.77 (1.49)	13.23 (1.55)	13.79 (1.55)	7–16
	**HA**	**MA**	**LA**	
**Exp 2**	31	54	39	
F:M	31:0	54:0	39:0	
Age	22.76 (2.31)	22.54 (2.36)	23.37 (2.36)	18–32
BDI-II	10.42 (5.28)	8.24 (5.19)	9.05 (5.19)	0–19
BVAQ	59.55(5.32)	46.70 (2.62)	38.35 (2.62)	28–71
AFF	21.65 (3.85)	18.05 (3.66)	15.13 (2.85)	9–27
COG	37.90 (5.70)	28.65 (5.08)	23.23 (3.69)	17–49
TDI	36.56 (3.21)	35.85 (3.10)	35.84 (2.56)	31.5–46
	**HA**	**MA**	**LA**	
**Exp 3**	118	156	109	
F:M	76:42	123:33	93:16	
Age	23.64 (4.97)	24.36 (5.49)	23.97 (4.46)	18–45
BDI-II	8.31 (4.93)	8.60 (5.01)	7.53 (5.11)	0–19
BVAQ	58.42 (4.97)	47.50 (2.84)	37.60 (3.86)	24–80
AFF	22.97 (4.42)	19.30 (3.86)	16.29 (3.63)	8–35
COG	35.46 (5.19)	28.20 (4.41)	21.31 (4.11)	12–54
SOS	27.34 (7.76)	29.58 (6.00)	31.29 (5.76)	4–44
AIO	2.04 (0.57)	2.22 (0.45)	2.36 (0.39)	0–3
VOIQ	54.52 (15.27)	56.65 (12.61)	59.03 (13.01)	16–80
OAS	109.80 (18.88)	114.39 (15.45)	120.71 (12.12)	34–152

HA = high alexithymia; MA = medium alexithymia; LA = Low alexithymia; F = females; M = males; SD = standard deviation; BVAQ = Bermond–Vorst Alexithymia Questionnaire; AFF = affective component of alexithymia; COG = cognitive component of alexithymia; TDI = total score of Sniffin’ Sticks test, BDI-II = Beck Depression Inventory-II, SOS = Social Odor Scale, AIO = Affective Importance of Odor scale, VOIQ = Vividness of Olfactory Imagery Questionnaire, OAS = Olfactory Awareness Scale.

### Bayesian results

*Exp 1*. TDI score analysis revealed a BF_01_ = 18.33 for the group variable Alexithymia Level, indicating strong evidence for the null hypothesis that general olfactory abilities do not vary by alexithymia level. Similar results were obtained for two subtests: threshold (group BF_01_ = 14.09) and discrimination (group BF_01_ = 16.59). For identification, results revealed indecisive to anecdotal evidence in support of the modulation of alexithymia level (BF_10_ = 2.30). See S1 Fig in S1 File.

*Exp 2*. The analyses revealed no evidence in support of the hypothesis for the moderation effect of the Alexithymia Level on the psychological attributes of odors: for each dependent variable (Intensity, Familiarity, Pleasantness of negative, positive, and neutral odors) the BF_10_ of the main effects and interactions were below 1. See S2 Fig in S1 File.

**Fig 1:**
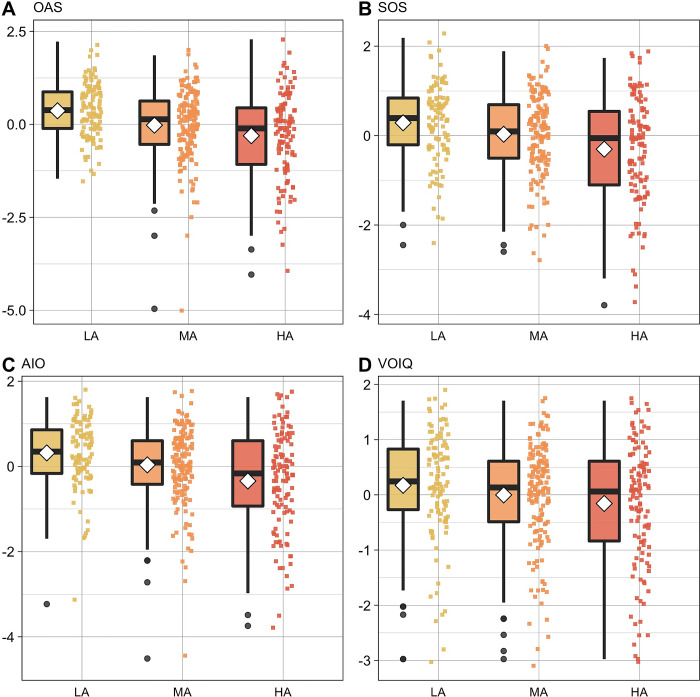
Data distribution of Experiment 3 by alexithymia groups (Low Alexithymia, LA; Medium Alexithymia, MA; High Alexithymia, HA) of A) OAS, B) SOS, C) AIO and D) VOIQ. Boxplots depict the median (horizontal black line) and quartile ranges of the distribution, whiskers indicate maximum and minimum values, colored dots represent data distribution.

*Exp 3*. We found very strong evidence in support of the hypothesis that alexithymia levels modulate odor awareness (OAS scores; group BF_10 =_ 6560.14) and that this modulation is affected by the BDI-II score (group + BDI-II BF_10_ = 752.88). Post-hoc tests showed that the HA group presents lower odor awareness than the LA (BF_10_ = 21543.334), but not the MA group (BF_10_ = 1.3). Moreover, LA present higher awareness than MA (BF_10_ = 52.79; Fig 1A). Similarly, we found very strong evidence in support of the hypothesis that the alexithymia levels modulate social odor awareness (SOS scores; group BF_10 =_ 390.94) and the affective importance given to odor (AIO scores; group BF_10 =_ 4742.25) and that this modulation is affected by the BDI-II score (SOS: group + BDI-II BF_10_ = 54.03; AIO: group + BDI-II BF_10_ = 700.66; Fig 1B). Post-hoc tests on the SOS scores showed that the HA group presents lower social odor awareness than the LA group (BF_10_ = 746.55) but only anecdotal or null evidence that there is a difference between the HA and MA groups (BF_10_ = 3.96) and between the LA and MA groups (BF_10_ = 1.76). Post-hoc tests on the AIO scores showed that the HA group presents lower affective importance for odors than the LA (BF_10_ = 8778.03) and MA groups (BF_10_ = 9.47) and anecdotal evidence that there is a difference between the LA and MA groups (BF_10_ = 3.18; Fig 1C). Finally, for VOIQ, the visual inspection of the q-q plot of the residuals showed a non-normal distribution. Therefore, extreme values were removed. The final model showed substantial evidence for the null hypothesis that olfactory imagery does not depend on alexithymia level (VOIQ scores; group BF_01_ = 5.01) and strong evidence for the null hypothesis once the modulation from BDI-II was considered (group + BDI-II BF_01_ = 29.44; Fig 1).

### Exploratory results

*Exp 1*. Models for TDI, Threshold and Discrimination scores did not contain any predictors. Results on Identification showed a main effect of Gender [F(1) = 4.50, *p* = 0.034]: male participants were better at identifying odors than female participants.

*Exp 2*. Results on the intensity ratings showed a main effect of the food/non-food factor [χ^2^ (1) = 18.82, p < .001], indicating that food odors were rated as more intense than non-food odors, and a significant interaction effect between the cognition subscale and the hunger rating [χ^2^ (1) = 4.80, *p* = 0.03].

**Fig 2:**
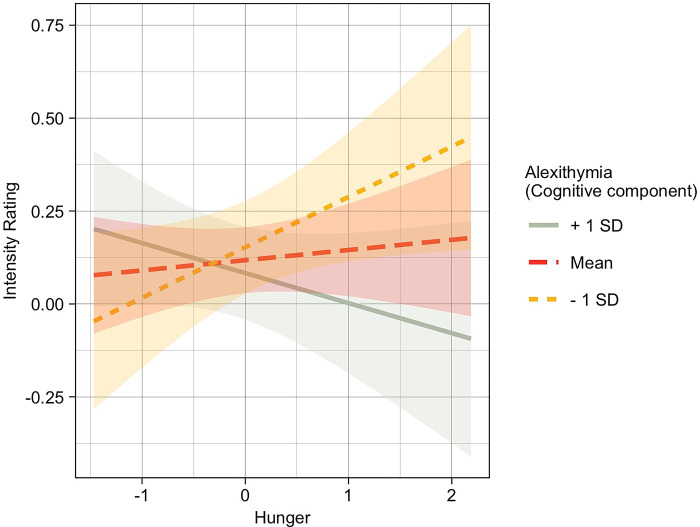
Fit lines of the Hunger*cognitive component interaction in the Intensity model for individuals with higher levels of cognitive alexithymic component (+1 SD), mean levels of cognitive alexithymic component, and low levels of cognitive alexithymic component (-1SD). Only among individuals with lower cognitive alexithymic component higher hunger levels predicted higher intensity ratings (orange line, *p* = 0.01).

Since the visual inspection of the q-q plot of the residuals indicated that they were not normally distributed, observations with values plus or minus 3 Median Absolute Deviation (MAD) from the median were removed (89 observations). The linear regression analysis was conducted again without these extreme values. The residuals were normally distributed and the new model similarly showed a main effect of the food/non-food factor [χ^2^ (1) = 12.38, p < .001], indicating that food odors were rated as more intense than non-food odors, and a significant interaction effect between the cognition subscale and the hunger rating [χ^2^ (1) = 5.12, *p* = 0.024]. Post-hoc tests indicated that among individuals with lower cognitive alexithymia (-1 SD), higher levels of hunger predicted higher intensity ratings of odors (*p* = 0.04), while among individuals with medium or higher (+1 SD) levels of alexithymia hunger levels did not affect intensity ratings (*p* = 0.54 and *p* = 0.21 respectively; Fig 2).

The analysis of the pleasantness ratings was performed separately for neutral, unpleasant and pleasant odors. The analysis of neutral odors showed a significant main effect of the food/non-food factor [χ^2^ (1) = 93.17, *p* < .001], reflecting that food odors were rated as more pleasant than non-food odors. For pleasant odors, the visual inspection of the q-q plot of the residuals showed a non-normal distribution. Therefore, extreme values were removed. The final model showed a significant main effect of the affective component of alexithymia of the BVAQ [χ^2^ (1) = 4.52, *p* = 0.033], indicating that the higher the level of affective alexithymia the least pleasant the odors rated, and a significant main effect of the food/non-food factor [χ^2^ (1) = 7.7396, *p* = 0.005], reflecting that food odors were rated as less pleasant than non-food odors. Results from unpleasant odors showed a significant main effect of the food/non-food factor [χ^2^ (1) = 47.80, *p* < .001], reflecting that non-food odors were rated as more pleasant than food odors, and a significant interaction effect between the cognition subscale and the hunger rating [χ^2^ (1) = 3.93, *p* = 0.047], however, no significant results were found in the post-hoc tests.

Results on familiarity ratings showed a main effect of the food/non-food factor [χ^2^ (1) = 288.14, *p* < .001], indicating that food odors were rated as more familiar than non-food odors. The triple interaction was significant [χ^2^ (1) = 4.75, *p* = 0.029], however, no significant results were found in the post-hoc tests.

*Exp 3*. Results for OAS showed a main effect of the COG factor [F(1) = 8.73, *p* = 0.003] and of the AFF factor [F(1) = 12.83, *p* < .001], indicating that higher levels of cognitive and affective alexithymia correlated with lower levels of odor awareness. Moreover, there was a main effect of gender [F(1) = 18.58, *p* < .001], age [F(1) = 7.67, *p* = 0.006], and smoking habits [F(1) = 4.47, *p* = 0.035], indicating that women presented higher levels of odor awareness compared to men, and a correlation between older age and higher odor awareness and fewer smoked cigarettes and higher odor awareness.

Results on the SOS scores showed a main effect of the COG factor [F(1) = 12.34, *p* < .001], indicating a correlation between higher levels of cognitive alexithymia and lower levels of social odor awareness. Moreover, there was a main effect of gender [F(1) = 5.69, *p* = 0.018], indicating that women presented higher levels of social odor awareness than men.

Results on the AIO scores showed a main effect of the COG factor [F(1) = 11.64, *p* < .001] and the AFF factor [F(1) = 14.89, *p* < .001], indicating a correlation between higher levels of cognitive and affective alexithymia and lower reported affective importance to odors. Moreover, there was a main effect of gender [F(1) = 8.04, *p* = 0.005], indicating that women reported giving higher affective importance to odors compared to men.

For VOIQ, the visual inspection of the q-q plot of the residuals showed a non-normal distribution. Therefore, extreme values were removed. The final model only showed a main effect of age [F(1) = 4.97, *p* = 0.026], indicating a correlation between older age and higher abilities in olfactory imagery.

## Discussion

In the present study, we conducted three experiments to clarify the relationship between olfactory processing and alexithymia. Broadly speaking, our results partially confirmed the registered hypotheses: as expected, individuals with a high level of alexithymia presented the same olfactory abilities and did not show differences in their rating of odors as pleasant, intense, and familiar compared to individuals with a low alexithymia level, while they reported lower levels of social and common odor awareness and a more indifferent attitude towards odors. However, we found that olfactory imagery did not vary between different alexithymia groups and that, when considered separately, the affective and cognitive components of alexithymia were differently associated to olfactory perception.

More specifically, Experiments 1 and 2 confirmed that individuals with alexithymia present intact basic odor perception. Experiment 2 extends this observation to the affective perceived qualities of odors. The results of Bayesian analyses on high, medium and low levels of alexithymia are in line with our previous results showing that alexithymia did not affect olfactory hedonics [44] and with previous data on subjective reports of emotional reactions [16,82–84]. On the other hand, our results are in contrast with previous studies showing reduced olfactory abilities in individuals with alexithymia [42], or higher perceived affective qualities of odors in alexithymia [43]. However, these two studies investigated the association between basic odor perception and alexithymia in individuals with other neurological or psychiatric conditions such as fibromyalgia syndrome and mood disorders making the results less comparable.

However, when we deeper explored the effects of the two dimensions of alexithymia (cognitive and affective) the results became more composite. First, individuals with a higher level of affective alexithymia rated the pleasant odors as less pleasant compared to individuals with a lower level of affective alexithymia. This result is in line with the evidence that alexithymic individuals present a lower tendency to experience positive emotions [19,85–87] and lower physiological reactivity to emotional stimuli [88–90]. The reason why only the affective component was implicated in our result may be explained by the fact that positive stimuli are less demanding in terms of cognitive emotion regulation abilities [91]. Moreover, since most of the odors presented in Experiment 2 were food odors, and seen the association between alexithymia and abnormal eating behaviors [92–94], we deeper explored these effects on the rating of various odor categories also considering the level of hunger. Interestingly, individuals with higher or medium levels of cognitive alexithymia (with difficulties processing emotions at the cognitive level and low abilities to identify, analyze, and verbalize one’s feelings) did not seem to rely on their physiological state to rate odor intensity, as did individuals with low levels of cognitive alexithymia, for whom being hungrier predicted higher odor intensity rating. This evidence is in line with the hypothesis that cognitive alexithymia is associated with difficulties in effective emotion regulation [95], leading to the use of maladaptive, externalizing behaviours, such as binge eating [93] or exercise addiction [95], as strategies for coping with negative mood [96–98]. An alternative hypothesis suggests that general alexithymia is associated with a decreased interoceptive awareness and with deficits in the ability to detect, monitor, and regulate internal bodily processes, such as hunger [99–101] and with alterations in food processing [92,102].

In Experiment 3, olfactory meta-cognitive abilities were tested. Both analyses confirmed our hypotheses that individuals with higher levels of alexithymia present lower awareness of the common and social odors in the environment and report being less guided and affected by good and bad odors. One possible explanation for this result may be that individuals with alexithymia exhibit reduced attentional allocation for emotional stimuli [9] and the tendency to externally oriented thinking style rather than focusing their attention on emotional [103]. Olfactory stimuli are intrinsically related to emotions: they can elicit strong emotional reactions and emotional biographical memories [23,104]. Individuals with alexithymia may allocate reduced attention also to olfactory stimuli to avoid the related emotional contents. Previous literature has shown that alexithymia can be a risk factor for affective disorders characterized by reduced olfactory performance (e.g., anxiety, depression, and eating disorders; [5,105,106]). Moreover, over time, reduced attention to olfactory stimuli could lead to a reduction in olfactory performance [52]. In line with these data, our results may suggest that reduced attention to olfactory stimuli in individuals with alexithymia may be seen as a risk factor for the development of affective disorders. Notably, this is the first study investigating how individuals with alexithymia perceive social odors. It has been suggested that social odors transfer social information that is critical to fostering relationships [107]. The lower interest towards social odors predicted by higher levels of cognitive alexithymia may aggravate dysfunctional interpersonal patterns, such as reduced social interaction and increased social rejections, which are frequently observed in individuals with alexithymia [108]. Future studies should explore this relationship more. We also found that olfactory imagery was not associated with cognitive or affective alexithymia levels. This result is in contrast with previous studies reporting general and olfactory imagery deficits in individuals with alexithymia [44,109–111]. This inconsistency may be the consequence of different experimental settings (paper and pencil vs online questionnaire) and it demands further investigations.

Finally, since previous literature has highlighted the role of the symptoms of depression in abnormal olfactory abilities [52,53,112], as well as the strong correlation between alexithymia and depression [105,113,114], in Experiments 2 and 3 we excluded individuals with a BDI-II score equal or higher than 20, a possible indication of moderate depression [51], and covaried the BDI-II scores of the included participants in the analyses. The results showed that the even low BDI-II scores interact with the alexithymia level in affecting the relationship with odors only in the meta-cognitive olfactory abilities: participants with higher BDI-II scores had a stronger correlation between alexithymia levels and odor awareness. These results support previous studies proposing that both depressive symptoms and alexithymia could affect olfactory processing [42,44], but they also suggest that depressive symptoms may specifically interact with alexithymia by reducing the level of awareness towards odors in the environment rather than basic olfactory abilities.

It is worth mentioning that a similar dissociation between perception and metacognition in alexithymia has been obtained in other domains. For example, previous research [115] on interoceptive abilities (the conscious perception and recognition of bodily signals [116]) has shown that higher alexithymia levels are associated with higher interoceptive accuracy and sensibility but with lower interoceptive awareness, suggesting a dissociation between interoceptive accuracy and awareness in alexithymia. Our data confirm and extend the results of these studies [115,117]: alexithymic individuals do not present difficulties in perceiving or reporting the presence of odors in the environment but rather have difficulty in interpreting their psychophysiological reactions to them as they are less able to reflect on the implications of these odors.

The present study has some limitations. First, the samples of the three experiments are mainly composed of women (81.3% of the sample in Exp 1, 100% of the sample in Exp 2, and 76.2% of the sample in Exp 3), so the effect of gender could not be tested in the present study. Previous studies have shown that men potentially present a higher level of alexithymia than women [118–120], even though the difference was relatively small [118]. Moreover, it has been reported that women tend to be more emotionally expressive [121,122], and are more prone to use emotion-regulation strategies [123] than men. Gender differences are also reported in relation to olfactory perception, with women being more sensitive of odors than men (even though the difference is small; [124]), using more extreme values to evaluate the hedonic characteristics of odors [125], and being more aware to odors in the environment [47,126]. For these reasons, we could hypothesise a stronger relationship between alexithymia and different aspects of olfactory processing in men, with alexithymic men showing less emotional reactivity to odors and being less aware to odors in the environment than alexithymic women. Future studies should try to replicate our results using more equally distributed sample sizes. Second, in Experiment 1, data on depressive symptoms were not available. This could be a limitation since previous studies have shown that depression is associated with lower olfactory abilities [53]; however, in Experiment 2 we replicated the analyses including the BDI-II scores as a covariate and the results were confirmed. Third, in Experiment 2, Body Mass Index (BMI) was not considered even though previous studies have shown that it affects the perceived hedonic qualities of odors [92,127]. Related to this point, future studies should also include a screening for the presence of eating disorders. Fourth, differently from Experiments 1 and 2, in Experiment 3 data were collected through online self-report questionnaires. This may have impacted the results, for example, because participants perceived fewer expectations than in the laboratory settings (e.g., [16]). Moreover, the German and Italian versions of the self-report questionnaires used in Experiment 3 were not validated versions but were translated by the authors for a previous study [47]. This aspect may limit the control over cultural and language differences across the groups. Despite these limitations, to our knowledge, the current study is the first that deeply investigates with pre-registered hypotheses and statistical analyses the relationship between olfactory processing and alexithymia.

In summary, since the olfactory system is tightly connected with the limbic system and odors can trigger strong emotional reactions and memories [33,104,128], this series of studies addressed the important question of whether individuals with alexithymia, who present abnormal emotional processing, have altered basic olfactory processing. The three experiments clarified that alexithymia does not affect basic olfactory abilities or the perception of intrinsic affective qualities of odors, but it does influence the extent to which individuals are aware of and are affected by odors in the environment. Moreover, our results support the view that alexithymia is a multidimensional construct, with the two dimensions (cognitive and affective) presenting dissociable underlying neural systems [59] and distinct emotional and physiological alterations [45,91]. The divergent results that we obtained especially on the affective perceived qualities of odors (Exp 2) between Bayesian results and the exploratory analyses performed on the cognitive and affective components of alexithymia may suggest that the odor rating task is more prone to reflect the association with one dimension over the other. Indeed, the odor valence rating seems to be predicted by the affective component while the odor intensity rating by the cognitive component. This emphasizes the need to consider these dimensions in future studies [9]. However, it is worth mentioning that previous studies highlighted some limitations on the reliability of the cognitive and affective subscales of the BVAQ [58] and on the way the BVAQ measures the emotionalizing aspect of alexithymia [103,129]. These critics suggest a partial overlap between the affective and cognitive subscales [58]. In light of these recent criticisms, our exploratory results on the distinction between affective and cognitive dimensions should be taken with caution.

Learning more about olfactory perception in individuals with alexithymia is important not only to have a better understanding of how alexithymia impacts the perception of hedonic stimuli coming from different sensory modalities [41] but also for possible treatments. For example, interventions that aimed to enhance the conscious perception of odors occurring in the environment have been found to lead to enhanced olfactory performance [130]. In clinical samples, it has been proposed that group therapy and CBT may be particularly suitable for patients with high levels of alexithymia [131]; with the insight that individuals with alexithymia may lack awareness of olfactory stimuli from their surroundings, it is possible to propose that these interventions may be particularly effective because they employ exposure, role-playing and nonverbal communication, guiding patients to describe what they are feeling while they experience emotional arousal during exposure exercises and improving their ability to be aware of their emotions and their surroundings [132]. These findings also provide evidence as to why mindfulness-based treatments of alexithymia have been found to be effective (for a review, see [133]), as at the core of mindfulness training there is the goal to increase attention to the present moment and the awareness of bodily sensation.

## Supporting information

S1 File (DOCX)
