# Supplementary material for: Alexithymia modulates the attitudes towards odors but not the olfactory abilities or the affective reactions to odors
Source: PLoS One. 2023 Jun 6;18(6):e0278496. doi: 10.1371/journal.pone.0278496 (PMC10243640; doi:10.1371/journal.pone.0278496)

**Supplementary material**

*Sniffin’ Sticks test*

The Sniffin’ Sticks test is a validated and widely used test composed of three subtests that assess three different olfactory functions (Hummel *et al.*, 2007). First, odor identification was measured by presenting 16 common odors, each paired with four verbal and visual descriptors in a multiple forced-choice format (three distractors and one target). Second, odor discrimination was assessed over 16 trials using a three alternative force choice (3AFC) task: for each trial, three pens were presented in random order, two containing the same odor and the third containing the target odor. Third, odor threshold was assessed for n-butanol again with a 3AFC task in which 16 triplets of pens were presented to the participants: one containing an odorous solution (target) and two blanks filled with the solvent (distractors). Sixteen dilutions of n-butanol were prepared in a geometric series starting from a 4% n-butanol solution. Participants had to identify the odor-containing pen. Reversal of the staircase was triggered when the odor was correctly identified in two successive trials. Threshold was defined as the mean of the last four of seven staircase reversals. In each sub-test, odors were presented in felt-tip pens filled with 4 ml of liquid odorants or odorants dissolved in propylene glycol. For the odor presentation, the cap was removed by the experimenter for approximately 3 s and the pen’s tip was placed approximately 2 cm in front of both nostrils.

**Supplementary results**

*Exploratory results*

Information of the final models:

*Exp1*. Models for TDI score and the Threshold sub-score did not contain any predictors (TDI: initial AIC = 542.41, final AIC= 533.68, *p* = 0.77; Threshold: initial AIC = 543.59, final AIC = 533.68, *p* = 0.91). Discrimination test model included only Language as random factor (initial AIC = 542.41, final AIC = 532.27, *p* = 1.0). The final model investigating Identification included only Language as random factor and Gender as a predictor (initial AIC = 536.47, final AIC = 530.48, *p* = 0.73, R^2^ = 0.07).

*Exp2*. Model for Intensity rating included the main effect of the factor food/non-food, the main effects and the interactions of the cognitive components of BVAQ, the hunger level, and participants’ ID as a random factor (initial AIC= 5545, final AIC= 5197.5, *p* = 1).

*Intensity ~ COG + food/non-food + Hunger + COG:Hunger + (1 | ID)*

Conditional R^2^ was equal to 0.30, and marginal R^2^ was equal to 0.02.

Model for Valence rating of neutral odors included the main effect of the food/non-food factor and participants’ ID as a random factor (initial AIC= 1308, final AIC= 1283.8, *p* = 0.73).

*Valence ~ food/non-food + (1 | ID)*

Conditional R^2^ was equal to 0.25, and marginal R^2^ was equal to 0.14.

Model for Valence rating of pleasant odors included the main effect of the affective components of the BVAQ, the main effect of food/non-food factor, and participants’ ID as a random factor (initial AIC= 1673.9, final AIC= 1645.3, *p* = 0.95).

*Valence ~ AFF + food/non-food + (1 | ID)*

Conditional R^2^ was equal to 0.25, and marginal R^2^ was equal to 0.02.

Model for Valence rating of unpleasant odors included the main effect of the food/non-food factor and the interaction between the cognitive component of the BVAQ and the hunger rating, and participants’ ID as a random factor (initial AIC= 1007.33, final AIC= 985.1, *p* = 0.81).

*Valence ~ food/non-food + COG*Hunger + (1 | ID)*

Conditional R^2^ was equal to 0.28, and marginal R^2^ was equal to 0.11.

Model for Familiarity rating included the triple interaction between the cognitive component of BVAQ, the food/non-food factor, and the hunger rating, and participants’ ID as a random factor (initial AIC= 5263.5, final AIC= 5246.2, *p* = 0.71).

*Familiarity ~ COG*food/non-food*Hunger + (1 | ID)*

Conditional R^2^ was equal to 0.25, and marginal R^2^ was equal to 0.12.

*Exp3*. Model for OAS scores included the main and interaction effects of the cognitive and the affective components of the BVAQ and the BDI-II scores, plus the main effects of age, gender, and smoking habits (initial AIC= 1044.5, final AIC= 1042.5, *p* = 1).

*OAS ~ COG*AFF*BDI + Age + Gender + Smoking*

R^2^ was equal to 0.16.

SOS model included the main effects of the cognitive and the affective components of the BVAQ, participants’ gender, and smoking habits (initial AIC= 1078.1, final AIC= 1067.4, *p* = 0.86).

*SOS ~ COG + AFF + Gender + Smoking*

R^2^ was equal to 0.76.

AIO model included the main effects of the cognitive and the affective components of the BVAQ, and participants’ gender (initial AIC= 1064.2, final AIC= 1052.5, *p* = 0.83).

*AIO ~ COG + AFF + Gender*

R^2^ was equal to 0.11.

VOIQ model included the main effect of participants’ age (initial AIC= 972.45, final AIC= 958.7, *p* = 0.79).

*VOIQ ~ Age*

R^2^ was equal to 0.013.

**Supplementary table 1s.** Zero-order correlations of Exp 1

|  | **BVAQ** | **B1** | **B2** | **B3** | **B4** | **B5** | **AFF** | **COGN** | **TDI** | **T** | **D** | **I** |
| --- | --- | --- | --- | --- | --- | --- | --- | --- | --- | --- | --- | --- |
| **BVAQ** | 1 | 0.67 | 0.46 | 0.56 | 0.46 | 0.53 | 0.61 | 0.82 | 0.03 | 0.05 | -0.01 | -0.02 |
| **B1** | 0.67 | 1 | 0 | 0.36 | 0.06 | 0.21 | 0.03 | 0.82 | 0.01 | -0.02 | 0 | 0.05 |
| **B2** | 0.46 | 0 | 1 | -0.09 | 0.15 | 0.08 | 0.82 | -0.01 | -0.06 | 0.02 | -0.09 | -0.1 |
| **B3** | 0.56 | 0.36 | -0.09 | 1 | 0.01 | 0.25 | -0.06 | 0.74 | 0.05 | 0.08 | -0.04 | 0.03 |
| **B4** | 0.46 | 0.06 | 0.15 | 0.01 | 1 | 0.16 | 0.69 | 0.09 | 0.16 | 0.09 | 0.2 | 0.03 |
| **B5** | 0.53 | 0.21 | 0.08 | 0.25 | 0.16 | 1 | 0.15 | 0.56 | -0.07 | -0.01 | -0.1 | -0.08 |
| **AFF** | 0.61 | 0.03 | 0.82 | -0.06 | 0.69 | 0.15 | 1 | 0.04 | 0.05 | 0.06 | 0.05 | -0.06 |
| **COGN** | 0.82 | 0.82 | -0.01 | 0.74 | 0.09 | 0.56 | 0.04 | 1 | 0 | 0.02 | -0.05 | 0.02 |
| **TDI** | 0.03 | 0.01 | -0.06 | 0.05 | 0.16 | -0.07 | 0.05 | 0 | 1 | 0.79 | 0.67 | 0.51 |
| **T** | 0.05 | -0.02 | 0.02 | 0.08 | 0.09 | -0.01 | 0.06 | 0.02 | 0.79 | 1 | 0.2 | 0.06 |
| **D** | -0.01 | 0 | -0.09 | -0.04 | 0.2 | -0.1 | 0.05 | -0.05 | 0.67 | 0.2 | 1 | 0.32 |
| **I** | -0.02 | 0.05 | -0.1 | 0.03 | 0.03 | -0.08 | -0.06 | 0.02 | 0.51 | 0.06 | 0.32 | 1 |

**Supplementary table 2s.** Zero-order correlations of Exp 2

|  | **BVAQ** | **B1** | **B2** | **B3** | **B4** | **B5** | **AFF** | **COGN** | **TDI** | **D** | **T** | **I** | **BD** |
| --- | --- | --- | --- | --- | --- | --- | --- | --- | --- | --- | --- | --- | --- |
| **BVAQ** | 1 | 0.69 | 0.4 | 0.6 | 0.43 | 0.69 | 0.56 | 0.88 | 0.15 | 0.16 | 0.09 | 0.01 | 0.2 |
| **B1** | 0.69 | 1 | -0.1 | 0.33 | 0.07 | 0.45 | -0.03 | 0.84 | 0.03 | 0.08 | -0.01 | 0.03 | 0.13 |
| **B2** | 0.4 | -0.1 | 1 | 0.09 | 0.08 | 0.06 | 0.81 | 0.01 | 0.22 | 0.05 | 0.17 | 0.08 | 0.12 |
| **B3** | 0.6 | 0.33 | 0.09 | 1 | 0 | 0.22 | 0.07 | 0.68 | 0.02 | 0.1 | 0.06 | -0.18 | 0.36 |
| **B4** | 0.43 | 0.07 | 0.08 | 0 | 1 | 0.26 | 0.65 | 0.14 | 0.12 | 0.12 | 0.07 | 0.03 | -0.26 |
| **B5** | 0.69 | 0.45 | 0.06 | 0.22 | 0.26 | 1 | 0.2 | 0.71 | 0.05 | 0.11 | -0.04 | 0.08 | 0.13 |
| **AFF** | 0.56 | -0.03 | 0.81 | 0.07 | 0.65 | 0.2 | 1 | 0.09 | 0.24 | 0.11 | 0.17 | 0.08 | -0.06 |
| **COGN** | 0.88 | 0.84 | 0.01 | 0.68 | 0.14 | 0.71 | 0.09 | 1 | 0.04 | 0.12 | 0 | -0.03 | 0.27 |
| **TDI** | 0.15 | 0.03 | 0.22 | 0.02 | 0.12 | 0.05 | 0.24 | 0.04 | 1 | 0.41 | 0.77 | 0.33 | 0.07 |
| **D** | 0.16 | 0.08 | 0.05 | 0.1 | 0.12 | 0.11 | 0.11 | 0.12 | 0.41 | 1 | -0.07 | 0 | -0.05 |
| **T** | 0.09 | -0.01 | 0.17 | 0.06 | 0.07 | -0.04 | 0.17 | 0 | 0.77 | -0.07 | 1 | -0.11 | 0.12 |
| **I** | 0.01 | 0.03 | 0.08 | -0.18 | 0.03 | 0.08 | 0.08 | -0.03 | 0.33 | 0 | -0.11 | 1 | -0.03 |
| **BDI** | 0.2 | 0.13 | 0.12 | 0.36 | -0.26 | 0.13 | -0.06 | 0.27 | 0.07 | -0.05 | 0.12 | -0.03 | 1 |

**Supplementary table 3s.** Zero-order correlations of Exp 3

|  | **BVAQ** | **AFF** | **COGN** | **B1** | **B2** | **B3** | **B4** | **B5** | **BDI** | **BOSS** | **AIO** | **VOIQ** | **OAS** |
| --- | --- | --- | --- | --- | --- | --- | --- | --- | --- | --- | --- | --- | --- |
| **BVAQ** | 1 | 0.61 | 0.85 | 0.7 | 0.47 | 0.53 | 0.45 | 0.69 | 0.11 | -0.24 | -0.29 | -0.13 | -0.28 |
| **AFF** | 0.61 | 1 | 0.1 | 0.06 | 0.83 | -0.05 | 0.68 | 0.22 | -0.11 | -0.13 | -0.23 | -0.06 | -0.2 |
| **COGN** | 0.85 | 0.1 | 1 | 0.84 | 0.04 | 0.7 | 0.11 | 0.72 | 0.21 | -0.21 | -0.21 | -0.12 | -0.22 |
| **B1** | 0.7 | 0.06 | 0.84 | 1 | 0 | 0.33 | 0.11 | 0.46 | 0.15 | -0.18 | -0.17 | -0.08 | -0.14 |
| **B2** | 0.47 | 0.83 | 0.04 | 0 | 1 | 0.01 | 0.15 | 0.11 | -0.03 | -0.05 | -0.13 | 0 | -0.1 |
| **B3** | 0.53 | -0.05 | 0.7 | 0.33 | 0.01 | 1 | -0.09 | 0.27 | 0.26 | -0.09 | -0.06 | -0.08 | -0.16 |
| **B4** | 0.45 | 0.68 | 0.11 | 0.11 | 0.15 | -0.09 | 1 | 0.25 | -0.16 | -0.17 | -0.23 | -0.09 | -0.23 |
| **B5** | 0.69 | 0.22 | 0.72 | 0.46 | 0.11 | 0.27 | 0.25 | 1 | 0.07 | -0.23 | -0.26 | -0.13 | -0.21 |
| **BDI** | 0.11 | -0.11 | 0.21 | 0.15 | -0.03 | 0.26 | -0.16 | 0.07 | 1 | 0.02 | -0.05 | -0.05 | -0.01 |
| **BOSS** | -0.24 | -0.13 | -0.21 | -0.18 | -0.05 | -0.09 | -0.17 | -0.23 | 0.02 | 1 | 0.49 | 0.37 | 0.64 |
| **AIO** | -0.29 | -0.23 | -0.21 | -0.17 | -0.13 | -0.06 | -0.23 | -0.26 | -0.05 | 0.49 | 1 | 0.33 | 0.6 |
| **VOIQ** | -0.13 | -0.06 | -0.12 | -0.08 | 0 | -0.08 | -0.09 | -0.13 | -0.05 | 0.37 | 0.33 | 1 | 0.49 |
| **OAS** | -0.28 | -0.2 | -0.22 | -0.14 | -0.1 | -0.16 | -0.23 | -0.21 | -0.01 | 0.64 | 0.6 | 0.49 | 1 |

**Supplementary Figure 1s.** Data distribution of Experiment 1 by alexithymia groups (Low Alexithymia, LA; Medium Alexithymia, MA; High Alexithymia, HA). Boxplots depict the median (horizontal black line) and quartile ranges of the distribution, whiskers indicate maximum and minimum values, colored dots represent data distribution.

**
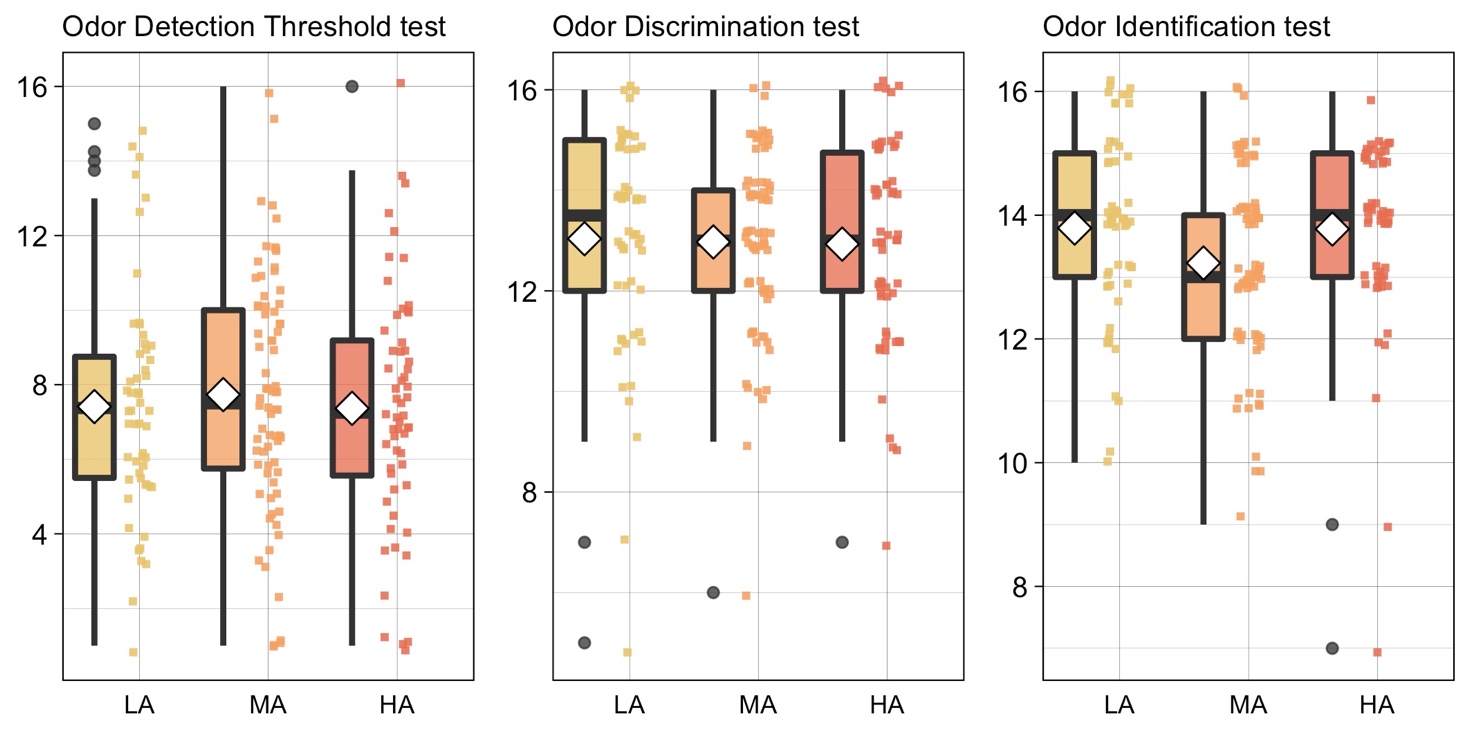
**

**Supplementary Figure 2s.** Data distribution of Experiment 2 by alexithymia groups (Low Alexithymia, LA; Medium Alexithymia, MA; High Alexithymia, HA). Boxplots depict the median (horizontal black line) and quartile ranges of the distribution, whiskers indicate maximum and minimum values, colored dots represent data distribution.


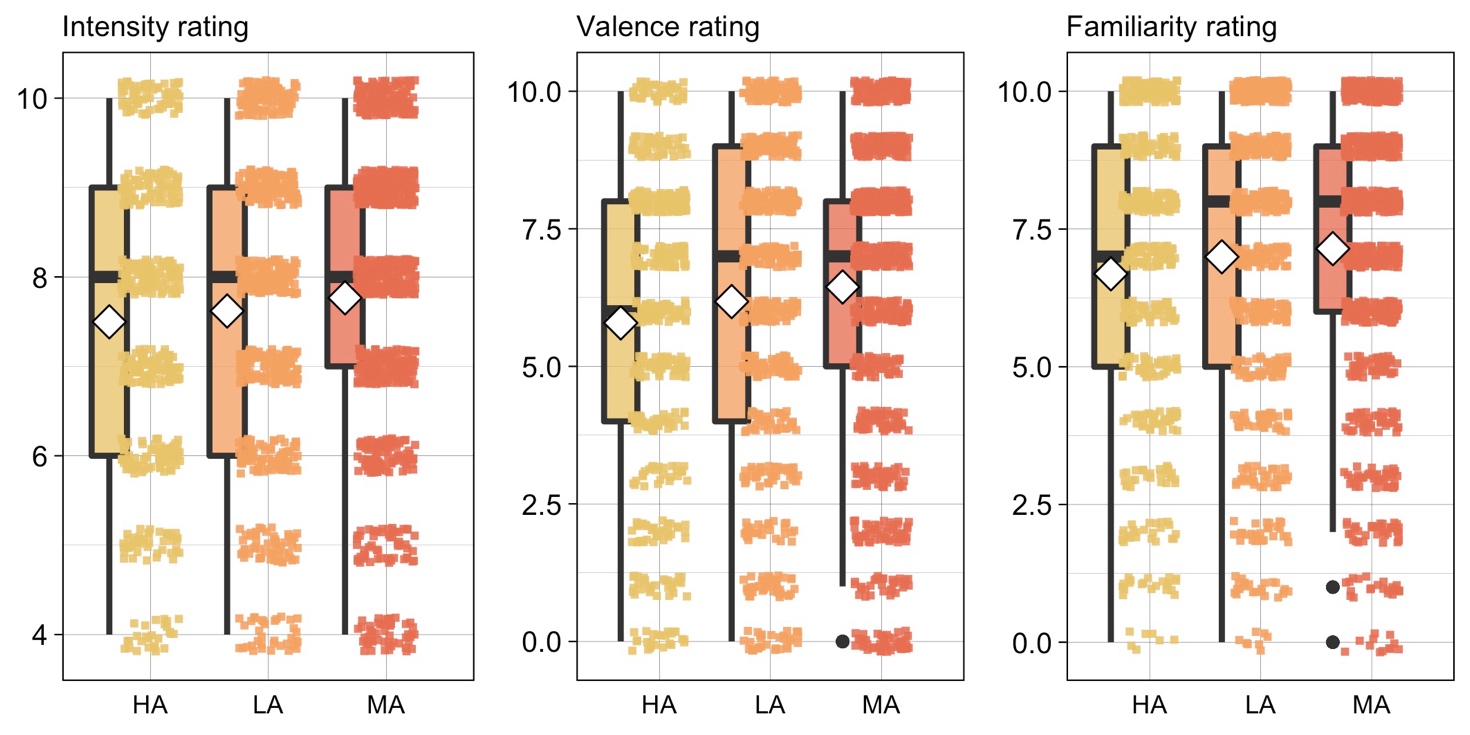

Supplement: S1 File — (DOCX) [file pone.0278496.s001.docx]
